# Assessment of Bio-physiological damages and cytological aberrations in cowpea varieties treated with gamma rays and sodium azide

**DOI:** 10.1371/journal.pone.0288590

**Published:** 2023-07-20

**Authors:** Aamir Raina, Younas Rasheed Tantray, Samiullah Khan

**Affiliations:** 1 Mutation Breeding Laboratory, Department of Botany, Aligarh Muslim University, Aligarh, India; 2 Botany Section, Women’s College, Aligarh Muslim University, Aligarh, India; 3 Botany Section, Central Ayurveda Research Institute, Jhansi, Uttar Pradesh, India; NBPGR: National Bureau of Plant Genetic Resources, INDIA

## Abstract

The assessment of mutagen induced biological damage forms an important study in determining the mutagenic potency and genotypic sensitivity, a vital aspect in mutation breeding programs. A prior assessment of lethal dose (LD50), mutagen induced biological damage (alterations in bio-physiological traits and frequency of cytological aberrations) is a prerequisite for determining an optimum mutagen dose in a successful mutation breeding experiment. Therefore, in a multi-year project of mutation breeding, two widely cultivated varieties of cowpea viz., Gomati VU-89 and Pusa-578, were treated with gamma (γ) rays and sodium azide (SA) doses. The results reflected a proportionate increase in bio-physiological damages with the increase in mutagenic doses and caused a substantial reduction in mean seed germination and seedling height. Different cytological aberrations such as cytomixis, univalents, chromosome stickiness, precocious separation, unequal separation, bridges, laggards, disturbed polarity, dyads, triads, and polyads were observed in both varieties. All the mutagen doses induced a broader spectrum of cytological aberrations with varying frequencies.

## 1. Introduction

*Vigna unguiculata* (L.) Walp. commonly known as cowpea is an important grain legume of the family Fabaceae, cultivated for food and forage in semi-arid and arid tropics that includes parts of Asia, Africa, Southern Europe, Central and South America, and the Southern United States [1, 2]. Africa is considered the centre of origin of cowpea [3] but the precise centre of domestication and diversity are still speculations [4, 5]. India is a secondary centre of diversity due to substantial genetic variability in the subcontinent [6]. The total cowpea production and the acreage have increased dramatically from 1.36 million tonnes (mt) from an area of 5.62 million hectares (mha) to 8.91 mt from an area of 14.91 mha in 1970 and 2021, respectively [7]. This increase in cowpea production and area harvested is due to climate change induced severe drought spells that resulted in the shifting of cultivation toward cowpea due to its drought tolerance [8]. Africa, especially the drier Savanna and Sahelian zones of West and Central Africa, contributes more than 95% of global cowpea production. Among countries, Nigeria is the largest cowpea producer and consumer, with 4.68 mha under cowpea cultivation and obtaining a production of 3.64 mt with an annual yield estimated at 777.6 kg/ha. Besides Nigeria, Niger is the next largest producer, with production estimated at 2.63 mt from 5.72 mha [7].

Cowpea is a multifunctional crop with immense potential to be an ideal crop in achieving food security in the era of climate change. Besides, it provides food for man, fodder for livestock, facilitates biological nitrogen fixation, enhances soil health, and is compatible with integrated farming systems [9]. Farmers and grain traders generate billions of revenue from millions of hectares of land under cowpea cultivation. Cowpea is popularly known as ‘poor man’s meat’ due to its high dietary protein (23.85 g), low fat (2.07 g), carbohydrates (59.64 g), dietary fibre (10.7 g), sugars (2.03 g), and several minerals and vitamins [10, 11]. The cowpea proteins are richer in tryptophan and lysine than cereals and lower in cysteine and methionine than animal proteins. Due to their high nutritional value, cowpea complements the protein-deficient cereal and tuber-based human diet. Cowpea is used at every stage of its growth [12]. The immature leaves and green pods are used as vegetables in India, Senegal, and African countries. The dry seeds are boiled and eaten as beans with cooked rice, and pearl millet [13]. Besides food values, cowpea also possess huge therapeutic importance in treating epilepsy, chest pain, hypertension, dysmenorrhea, migraines, and urinary shistomiasis [14–18].

Despite being a multifunctional crop, cowpea is among the neglected grain legumes, and very little attention has been given to its genetic improvement. This is reflected in the low yielding potential of cowpea compared to other legumes. Working on the genetic upgradation of the yielding potential of cowpea, we initiated a multi-year project on the mutagenesis and selection of high yielding mutant lines. We have successfully developed several novel high yielding mutant lines and worked out the mutagenesis protocol as a breeding strategy for improving cowpea. In the present study, we have focussed on optimizing γ rays and SA doses that could be used to improve existing cowpea cultivars.

Mutation breeding is among the successful breeding strategies with the least ethical and social criticism, unlike genetic engineering, which was employed to improve the cowpea varieties [19]. Mutation breeding enjoys widespread acceptance and has delivered much better results in developing thousands of mutant varieties. Mutation breeding equips the breeder with the scope of improving a single trait in an otherwise outstanding variety without compromising the genetic constitution [20]. Mutagens interact in numerous ways with the genes and create a wide range of alterations such as substitution, deletion, chromosome fragmentations, single strand, and double-strand DNA breaks [21]. Generally, chemical mutagens induce silent or missense mutations, and physical mutagens induce single-nucleotide insertions, substitutions, inversions, translocations, and short deletions [22]. The nature of induced mutation depends on the plant material treated, mutagen type, and dose and duration chosen. Unlike other legumes, cowpea is least exploited in mutation breeding programs. A lot of ambiguity exists in selecting mutagen doses that can be used to improve cowpea’s agronomic traits. Working on this knowledge gap, we have substantially improved the literature body by providing more details on the mutagen doses. Several aspects, such as mutagenic potency, treatment duration, and selection of plant material are important in the successful conduct of mutation breeding. It is also important to mention that mutagenic potency depends on the extent of biological damage and frequency of desirable mutations. The biological damage can be assessed by analyzing the spectrum and frequency of cytological aberrations in different doses of mutagens. In addition, biological damage can also be assessed by recording the germination percentage and height of seedlings. In the earlier publications, we presented detailed aspects of germination percentage and plant survival in all the mutagen doses [23]. In the present study, we will focus only on the doses of γ rays and SA that yielded a high frequency of mutations and relatively low biological damage. Generally increasing mutagenic doses augments both the frequency of mutations and biological damages; therefore, selecting appropriate mutagen for maintaining a balance between the frequency of desirable mutations and the extent of bio-physiological and cytological damages is necessary [24]. Therefore, optimizing a mutagen dose and duration and selecting appropriate plant material is crucial in achieving maximum mutation frequency with less biological damage [25].

The γ rays and SA were chosen as mutagens as none of the workers reported an optimum dose that could be used to improve the cowpea varieties [26]. The γ rays interact with tissues, hydrolyse water molecules and eventually lead to the build-up of highly reactive free radicals. These free radicals dislocate DNA-DNA cross-links which in turn induce a wide range of random mutations [27]. On the other hand, SA interacts with DNA via an organic metabolite, β-azidoalanine moiety [N3–CH2–CH(–NH2)–COOH], that induces AT→GC base pair transition [28, 29].

## 2. Materials and methods

### 2.1. Experimental materials and seed treatment

We obtained dry and healthy seeds of two cowpea varieties viz., Gomati VU-89 and Pusa-578, from ICAR-National Bureau of Plant Genetic Resources, New Delhi. First batch of 300 seeds were irradiated with 100 to 1000 Gy of γ rays with an interval of 100 Gy. Seeds were treated with γ rays in a Gamma chamber 900 ^60^Co (NBRI, Lucknow) at a dose rate of 11.58 Gy/min. A separate batch of 300 seeds each was soaked in distilled water for six hours and then immersed in beakers containing 0.01 to 0.1% of SA with an interval of 0.01% SA for nine hours at the Department of Botany, Aligarh Muslim University (AMU), Aligarh, India. Already irradiated seeds were treated with SA, and doses were labelled as 100 Gy+0.01%, 200 Gy+0.02%, 300 Gy+0.03%, 400 Gy+0.04%, 500 Gy+0.05%, 600 Gy+0.06%, 700 Gy+0.07%, 800 Gy+0.08%, 900 Gy+0.09% and 1000 Gy+0.1% of γ rays+SA. The study showed that doses beyond 400 Gy γ rays, 0.04% SA and 400 Gy+0.04% SA induced more than 50% reduction in plant survival [23]. Therefore, only the first four individual and combination treatments were advanced to future generations.

#### 2.1.1. Field methodology

In mid-April 2014, we chose 300 seeds for the control set and 300 seeds treated with each mutagen dose employed individually and in combination. Seeds were sown in the agriculture farm of AMU, Aligarh, in a randomized complete block design. All the M_1_ plants were harvested separately, and seeds were stored to raise subsequent generations.

### 2.2. Germination percentage

Seed germination was calculated using the following formula.


$$Germination\;(\%)=\frac{No.\;of\;seeds\;germinated}{No.\;of\;seeds\;sown}\;x\;100$$

### 2.3. Lethal dose 50

The data generated on seed germination were used to evaluate the LD50 doses for each mutagenin both Gomati VU-89 and Pusa-578 varieties. We calculated LD50 using probit analysis in OPSTAT software [30, 31].

### 2.4. Seedling growth parameters

Seedling height was measured after 14 days of seed sowing by recording the length of roots and shoots in treated and untreated seedlings. Mutagen induced seedling injury was measured using the following formula.


$$Seedling\;Injury\;(\%)=\frac{Seedling\;height\;in\;control-Seedling\;height\;in\;treatment}{Seedling\;height\;in\;control}\;x\;100$$

### 2.5. Pollen fertility

During flowering, mature floral buds were collected in the early morning hours, fixed in 70% ethanol, and kept at 4°C for further analysis. Anthers were crushed in a glycerol and acetocarmine mixture (1:1) to assess pollen fertility. Uniformly stained pollen grains were fertile, whereas unstained pollen grains were considered sterile. Pollen fertility was calculated using the following formula.


$$Pollen\;Fertility\;(\%)=\frac{Pollen\;fertility\;in\;control-Pollen\;fertility\;in\;treatment}{Pollen\;fertility\;in\;control}x\;100$$

### 2.6. Cytological observations: Meiotic analysis

In June–July 2014, immature floral buds from 30 M_1_ plants selected in each treated and control plant were fixed in Carnoy’s fixative (acetic acid: chloroform: ethanol, in the volume ratio of 1:3:6) for 24 hrs and subsequently stored in 70% ethanol at 4°C. Complete observations of chromosome counts and meiotic aberrations were made from slides of meiocytes, prepared by crushing and squashing of immature anthers in 1% acetocarmine. Pollen mother cells (PMCs) were examined for meiotic aberrations at prophase I, metaphase I (M I), anaphase I (A I), telophase I (T I), and sporads. Photomicrographs of PMCs showing chromosome counts and meiotic aberrations were obtained from Leica Qwin ver. V. 2.3 Digital Imaging System and a Nikon 80*i* Digital Imaging System.

## 3. Results

### 3.1. Bio-physiological traits

#### 3.1.1. Germination percentage

**Fig 1:**
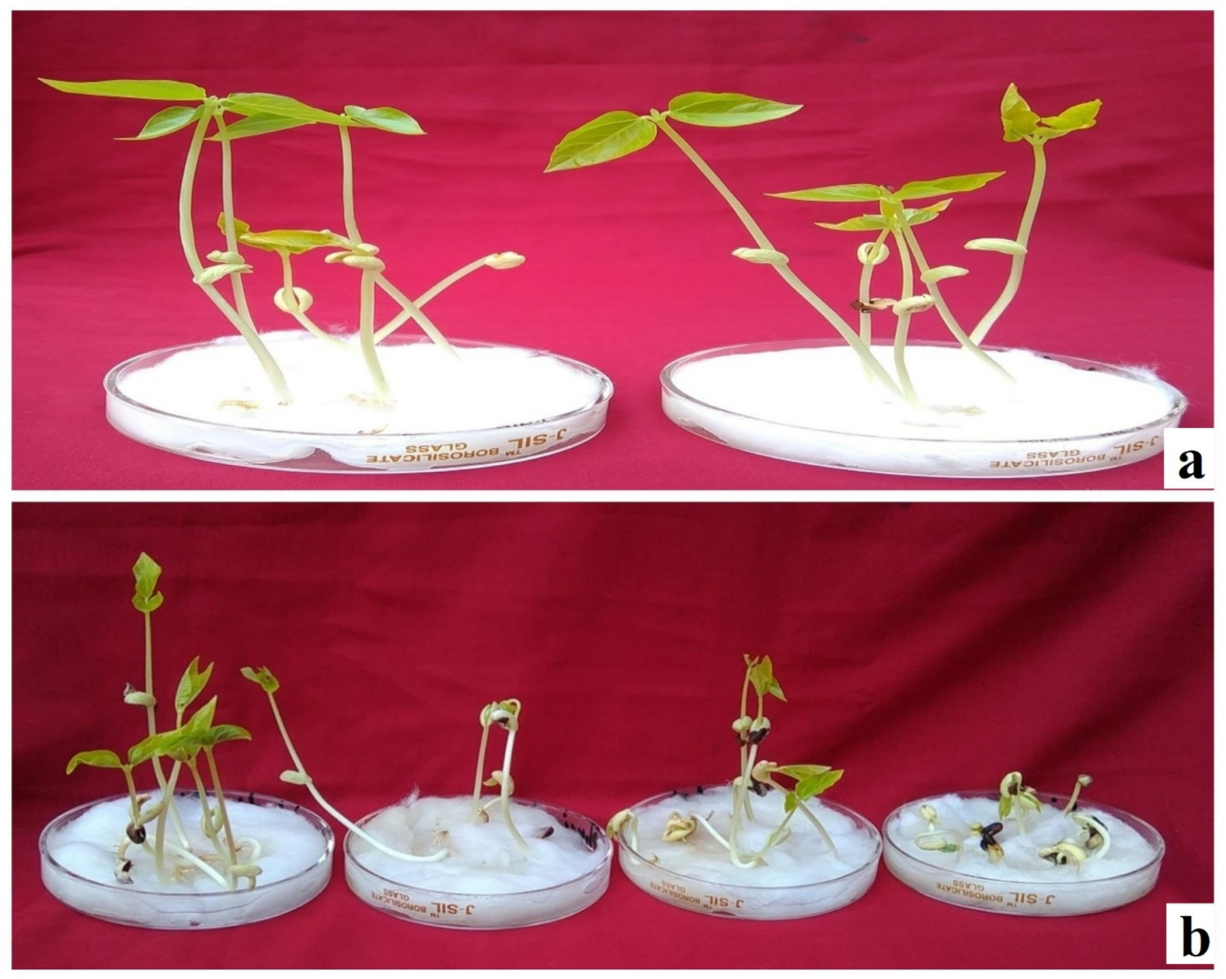
a: Seed germination in control; b: Progressive decrease in seed germination with increased mutagen concentration from 100 Gy to 400 Gy gamma rays in var. Gomati VU-89.

The seed germination percentage showed a progressive decline with the increasing dose of γ rays and SA (Table 1). In the control populations, the seed germination was recorded as 92.67 and 90.67% in the varieties Gomati VU-89 and Pusa-578, respectively (Fig 1A). Combined mutagens induced more germination inhibition followed by γ rays and SA treatments in both varieties (Fig 1B). While comparing with control, a statistically significant decrease in germination percentage in all the mutagenized population of both varieties was recorded. The maximum germination inhibition percentage was recorded as 20.86 and 24.63% in 400 Gy+0.04% SA treatment in Gomati VU-89 and Pusa-578 varieties, respectively. The results revealed that lower dose of both γ rays (100 Gy) and SA (0.01%) were less detrimental in inducing biological damage compared to higher mutagen doses.

**Table 1:** Effects of gamma rays, sodium azide and their combinations on mean seed germination and germination inhibition in two cowpea varieties. The data is presented as percent (n = 3) and standard error (SE). Mean values with the same letters are not significant at a 5% level of significance, based on Duncan’s multiple range test (DMRT).

Treatments	var. Gomati VU-89		var. Pusa-578	
	Germination Mean±SE	Inhibition (%)	Germination Mean±SE	Inhibition (%)
Control	92.67^a^±044	--	90.67^a^±0.45	--
G1	87.00^b^±0.41	6.12	83.67^b^±0.42	7.72
G2	85.00^c^±0.45	8.27	81.67^cd^±0.39	9.93
G3	81.67^d^±0.39	11.87	76.67^e^±0.51	15.44
G4	80.00^e^±0.19	13.67	73.33^fg^±0.32	19.12
S1	86.67^b^±0.18	6.47	83.33^bc^±0.51	8.09
S2	84.67^c^±0.42	8.63	81.33^d^±0.67	10.29
S3	82.00^d^±0.61	11.51	76.33^e^±0.61	15.81
S4	79.33^e^±0.34	14.39	73.00^g^±0.48	19.49
G1+S1	85.67^c^±0.31	7.55	80.67^d^±0.48	11.03
G2+S2	81.67^d^±0.17	11.87	75.00^ef^±0.74	17.28
G3+S3	78.33^f^±0.45	15.47	70.00^h^±1.51	22.79
G4+S4	73.33^g^±0.32	20.86	68.33^h^±0.58	24.63

G1 = 100 Gy γ rays, G2 = 200 Gy γ rays, G3 = 300 Gy γ rays, G4 = 400 Gy γ rays, S1 = 0.01% SA, S2 = 0.02% SA, S3 = 0.03% SA, S4 = 0.04% SA, G1+S1 = 100 Gy γ rays+0.01% SA, G2+S2 = 200 Gy γ rays+0.02% SA, G3+S3 = 300 Gy γ rays+0.03% SA, G4+S4 = 400 Gy γ rays+0.04% SA

#### 3.1.2. Evaluation of LD50

**Fig 2:**
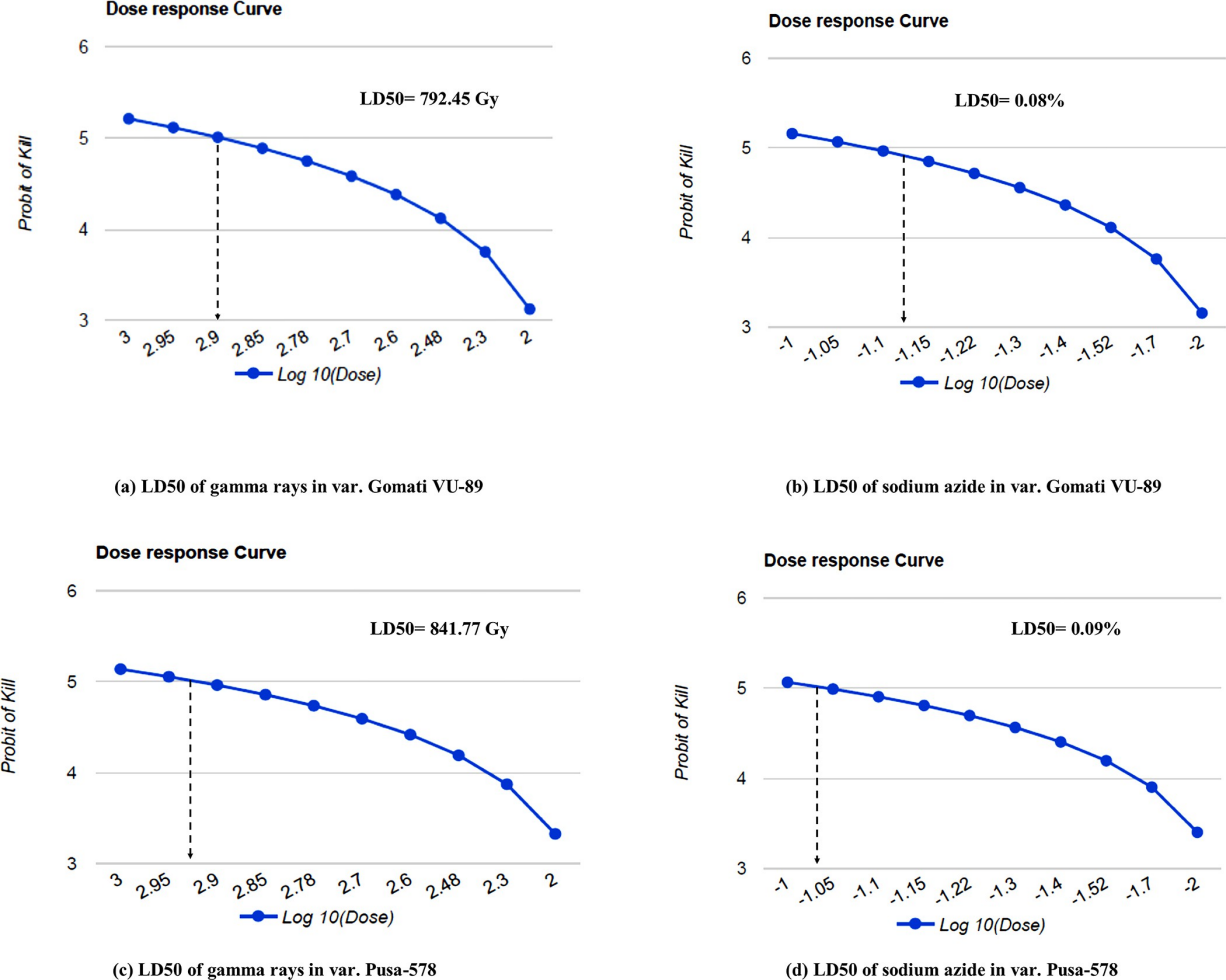
a: Dose response curve and LD50 of gamma rays in var. Gomati VU-89, b: Dose response curve and LD50 of sodium azide in var. Gomati VU-89, c: Dose response curve and LD50 of gamma rays in var. Pusa-578, d: Dose response curve and LD50 of sodium azide in var. Pusa-578.

The LD50 of γ rays was calculated as 792.45 and 841.77 Gy in the variety Gomati VU-89 and Pusa-578, respectively (Fig 2). In SA treated plants, the LD50 was calculated as 0.08 and 0.09% SA in the variety Gomati VU-89 and Pusa-578, respectively (Fig 2). The results of probit analysis revealed that lower doses of both γ rays and SA were less detrimental and could be considered as optimum doses for mutation breeding in cowpea.

#### 3.1.3. Seedling growth parameters

**Fig 3:**
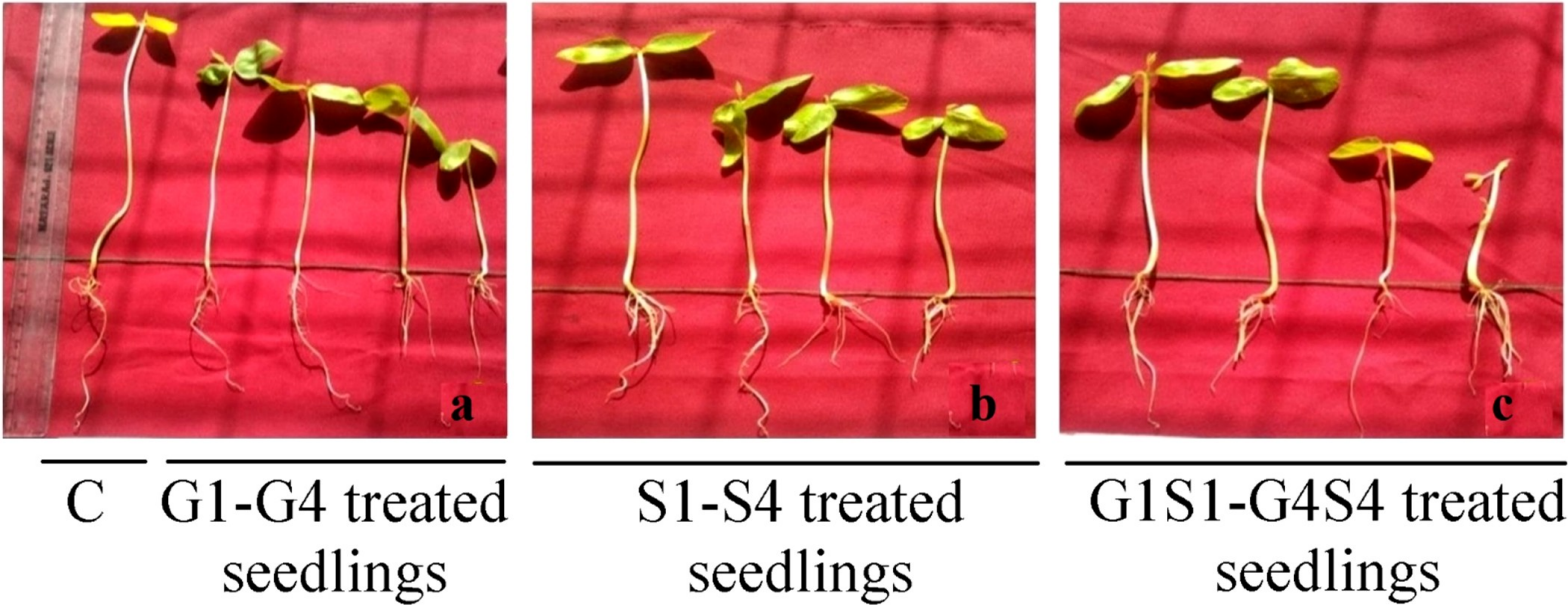
Variations in shoot, root and total seedling height in a: control and gamma treated seeds (G1-G4), b: sodium azide treated seeds (S1-S4) and c: combined mutagen treated seeds (G1S1-G4S4). G1 (100 Gy), G2 (200 Gy), G3 (300 Gy), G4 (400 Gy), S1 (0.01% SA), S2 (0.02% SA), S3 (0.03% SA), S4 (0.04% SA), 100 Gy γ rays+0.01% SA (G1S1), 200 Gy γ rays+0.02% SA (G2S2), 300 Gy γ rays+0.03% SA (G3S3) 400 Gy γ rays+0.04% SA (G4S4).

Mutagens induced a progressive decrease in mean seedling height with the increasing doses of mutagens (Fig 3). It is more appropriate to discuss the results of seedling height into separate subheadings as mentioned below:

#### 3.1.4. Root length

**Fig 4:**
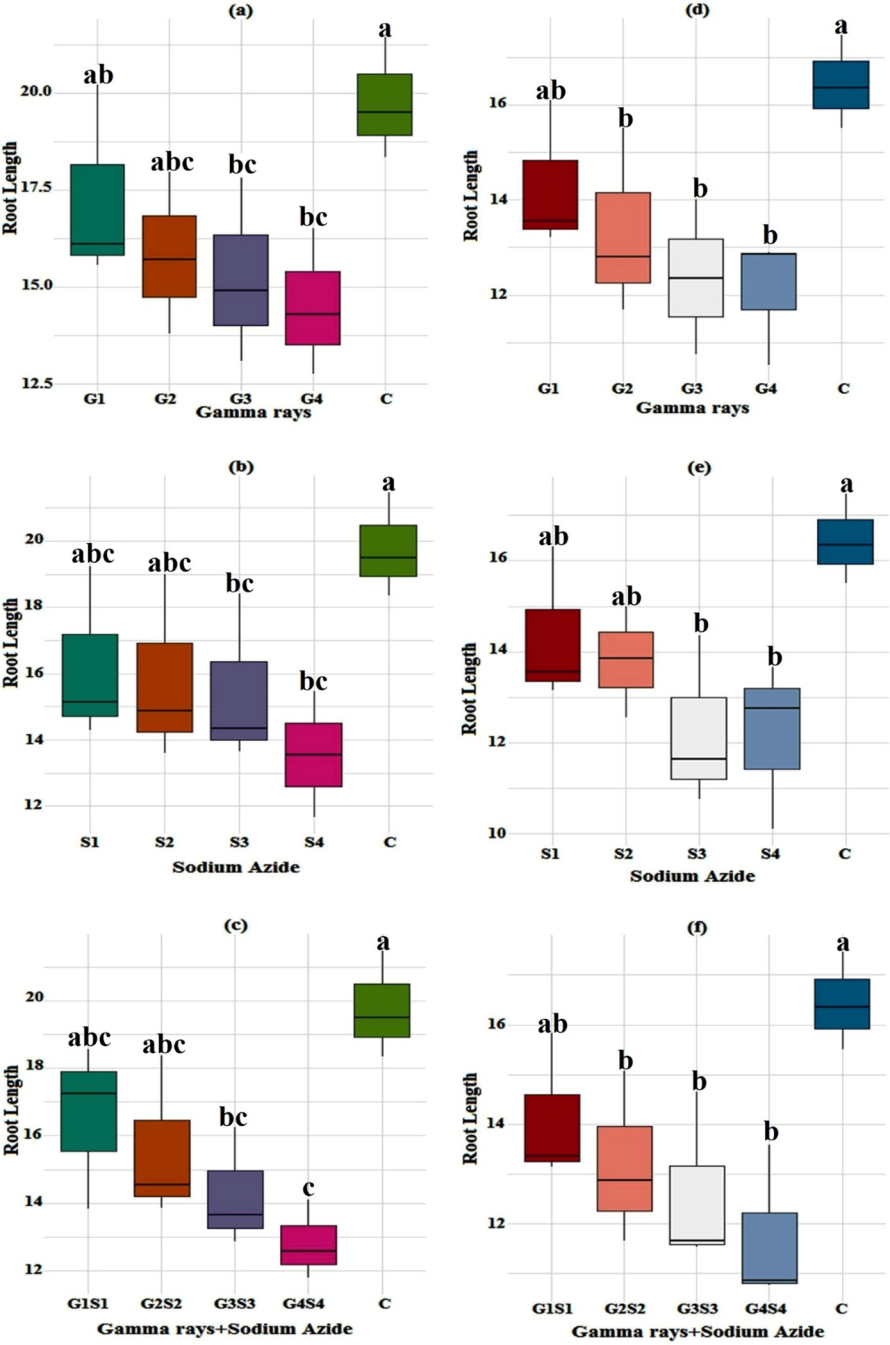
Effect of gamma radiations, sodium azide, and their combinations on mean root length (Mean±SE) (n = 30) in var. Gomati VU-89 (a-c) and var. Pusa-578 (d-f). G1 (100 Gy), G2 (200 Gy), G3 (300 Gy), G4 (400 Gy), S1 (0.01% SA), S2 (0.02% SA), S3 (0.03% SA), S4 (0.04% SA), 100 Gy γ rays+0.01% SA (G1S1), 200 Gy γ rays+0.02% SA (G2S2), 300 Gy γ rays+0.03% SA (G3S3) 400 Gy γ rays+0.04% SA (G4S4). #Means followed by the same letter is not different at 5% level of significance, based on the DMRT.

The mean root length in the control population was measured as 19.77 and 16.44 cm in the var. Gomati VU-89 and var. Pusa-578, respectively. In the var. Gomati VU-89, the maximum length of roots, was recorded as 17.29 cm in 100 Gy treatment. In the var. Pusa-578, the maximum length of roots was recorded as 14.33 cm in 0.01% SA treatment (Fig 4)

#### 3.1.5. Shoot length

**Fig 5:**
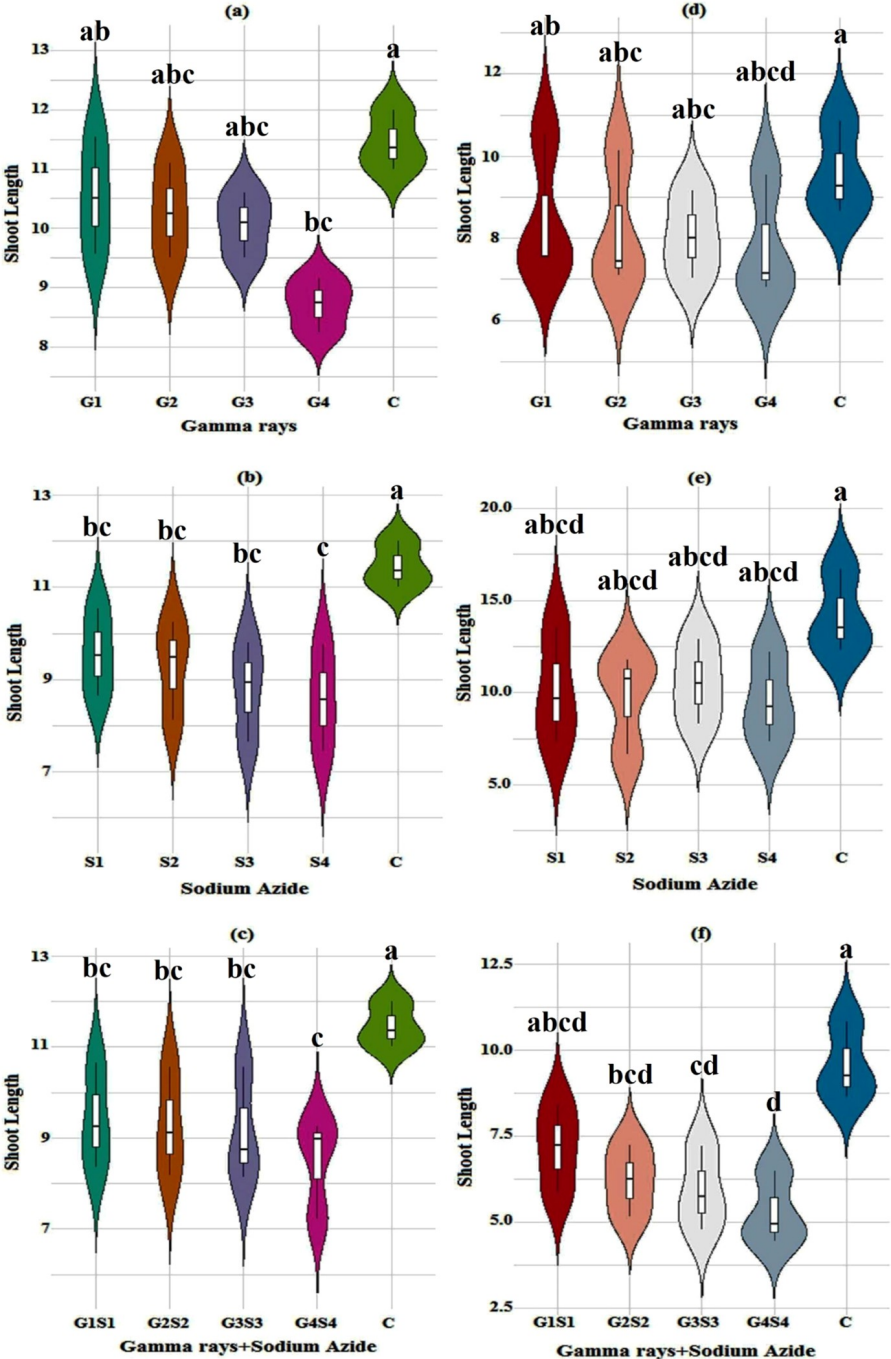
Effect of gamma radiations, sodium azide, and their combinations on mean shoot length (Mean±SE) (n = 30) in var. Gomati VU-89 (a-c) and var. Pusa-578 (d-f). G1 (100 Gy), G2 (200 Gy), G3 (300 Gy), G4 (400 Gy), S1 (0.01% SA), S2 (0.02% SA), S3 (0.03% SA), S4 (0.04% SA), 100 Gy γ rays+0.01% SA (G1S1), 200 Gy γ rays+0.02% SA (G2S2), 300 Gy γ rays+0.03% SA (G3S3) 400 Gy γ rays+0.04% SA (G4S4). #Means followed by the same letter is not different at 5% level of significance, based on the DMRT.

The mean shoot length in the control population was measured as 11.45 and 9.59 cm in the var. Gomati VU-89 and var. Pusa-578, respectively. In the var. Gomati VU-89, the maximum length of the shoots was recorded as 10.53 cm in 100 Gy treatment. In the var. Pusa-578, the maximum length of shoots was recorded as 8.54 cm in 100 Gy treatment (Fig 5).

#### 3.1.6. Seedling height

**Fig 6:**
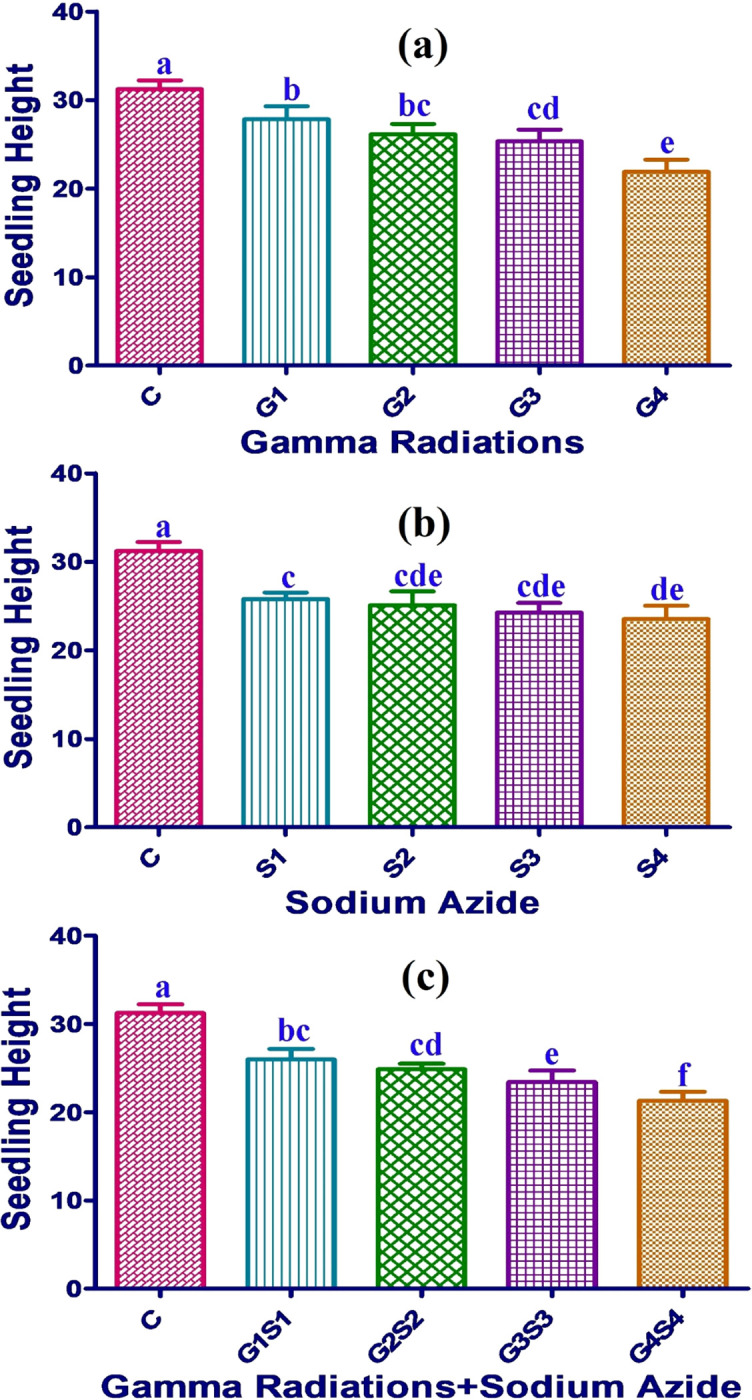
Effect of gamma radiations (a), sodium azide (b), gamma radiations+sodium azide (c) on mean seedling height (Mean±SE) (n = 30) in var. Gomati VU-89. G1 (100 Gy), G2 (200 Gy), G3 (300 Gy), G4 (400 Gy), S1 (0.01% SA), S2 (0.02% SA), S3 (0.03% SA), S4 (0.04% SA), 100 Gy γ rays+0.01% SA (G1S1), 200 Gy γ rays+0.02% SA (G2S2), 300 Gy γ rays+0.03% SA (G3S3) 400 Gy γ rays+0.04% SA (G4S4). #Means followed by the same letter is not different at 5% level of significance, based on the DMRT.

**Fig 7:**
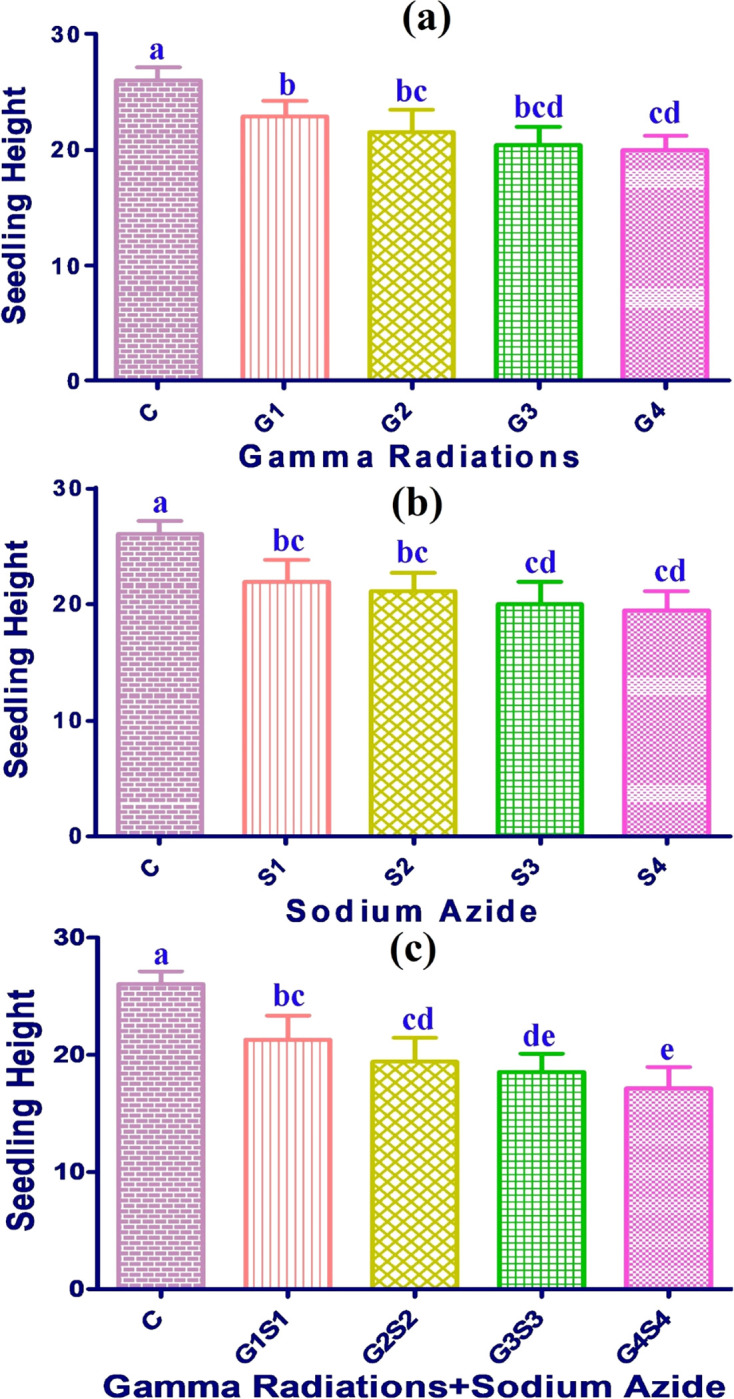
Effect of gamma radiations (a), sodium azide (b), gamma radiations+sodium azide (c) on mean seedling height (Mean±SE) (n = 30) in var. Pusa-578. G1 (100 Gy), G2 (200 Gy), G3 (300 Gy), G4 (400 Gy), S1 (0.01% SA), S2 (0.02% SA), S3 (0.03% SA), S4 (0.04% SA), 100 Gy γ rays+0.01% SA (G1S1), 200 Gy γ rays+0.02% SA (G2S2), 300 Gy γ rays+0.03% SA (G3S3) 400 Gy γ rays+0.04% SA (G4S4). #Means followed by the same letter is not different at 5% level of significance, based on the DMRT.

In control populations, the total seedling height was measured as 31.23 and 26.03 cms in Gomati VU-89 and Pusa-578 varieties, respectively. Maximum seedling height was recorded as 27.82 and 22.83 cm in 100 Gy treatment in the var. Gomati VU-89 and var. Pusa-578, respectively. Combination treatments showed a higher decline in the seedling height, followed by SA and γ rays treatments (Figs 6 and 7).

#### 3.1.7. Seedling injury

**Fig 8:**
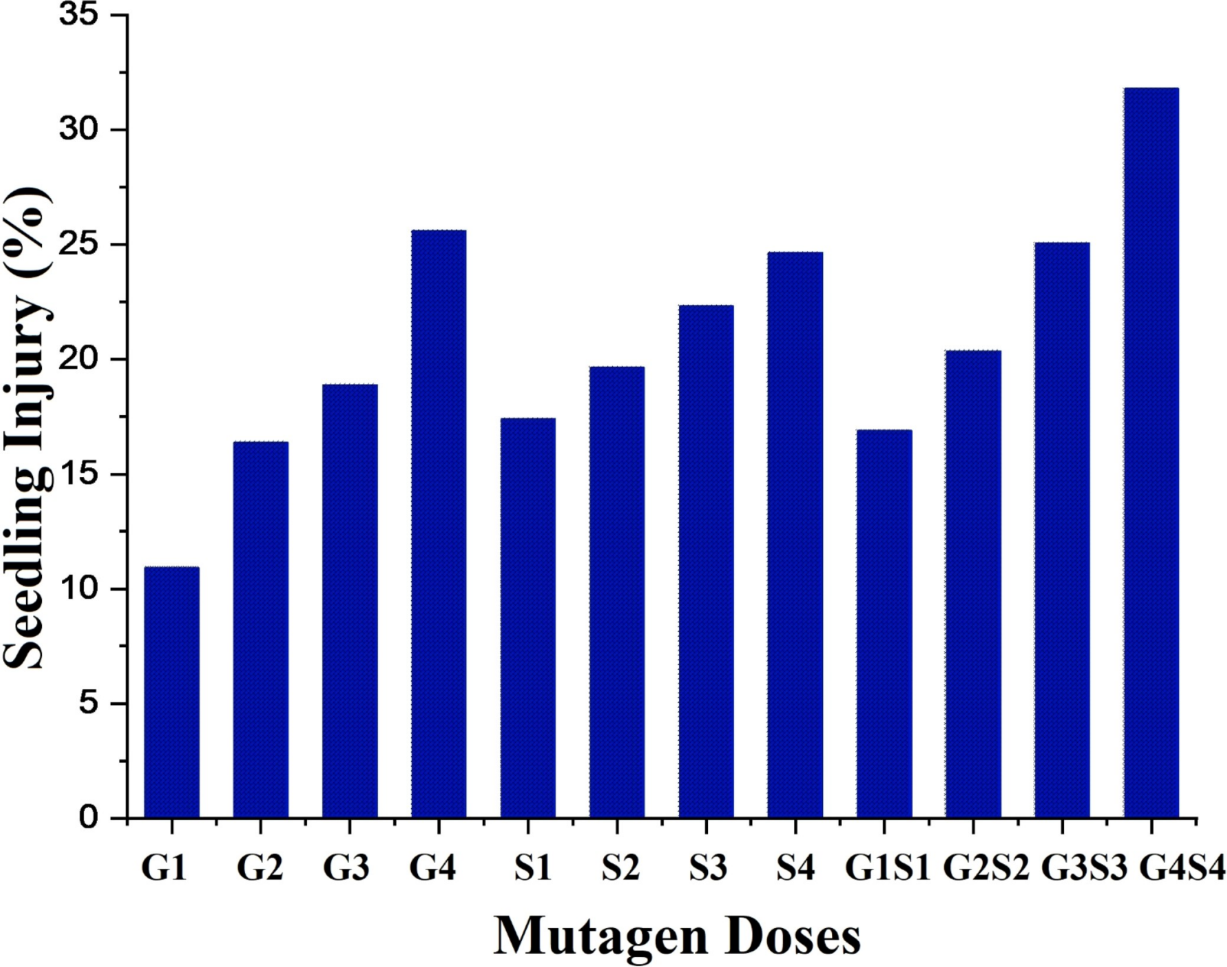
Effect of different doses of gamma rays, sodium azide and their combinations on seedling injury (%) in var. Gomati VU-89. G1 = 100 Gy γ rays, G2 = 200 Gy γ rays, G3 = 300 Gy γ rays, G4 = 400 Gy γ rays, S1 = 0.01% SA, S2 = 0.02% SA, S3 = 0.03% SA, S4 = 0.04% SA, G1+S1 = 100 Gy γ rays+0.01% SA, G2+S2 = 200 Gy γ rays+0.02% SA, G3+S3 = 300 Gy γ rays+0.03% SA, G4+S4 = 400 Gy γ rays+0.04% SA.

**Fig 9:**
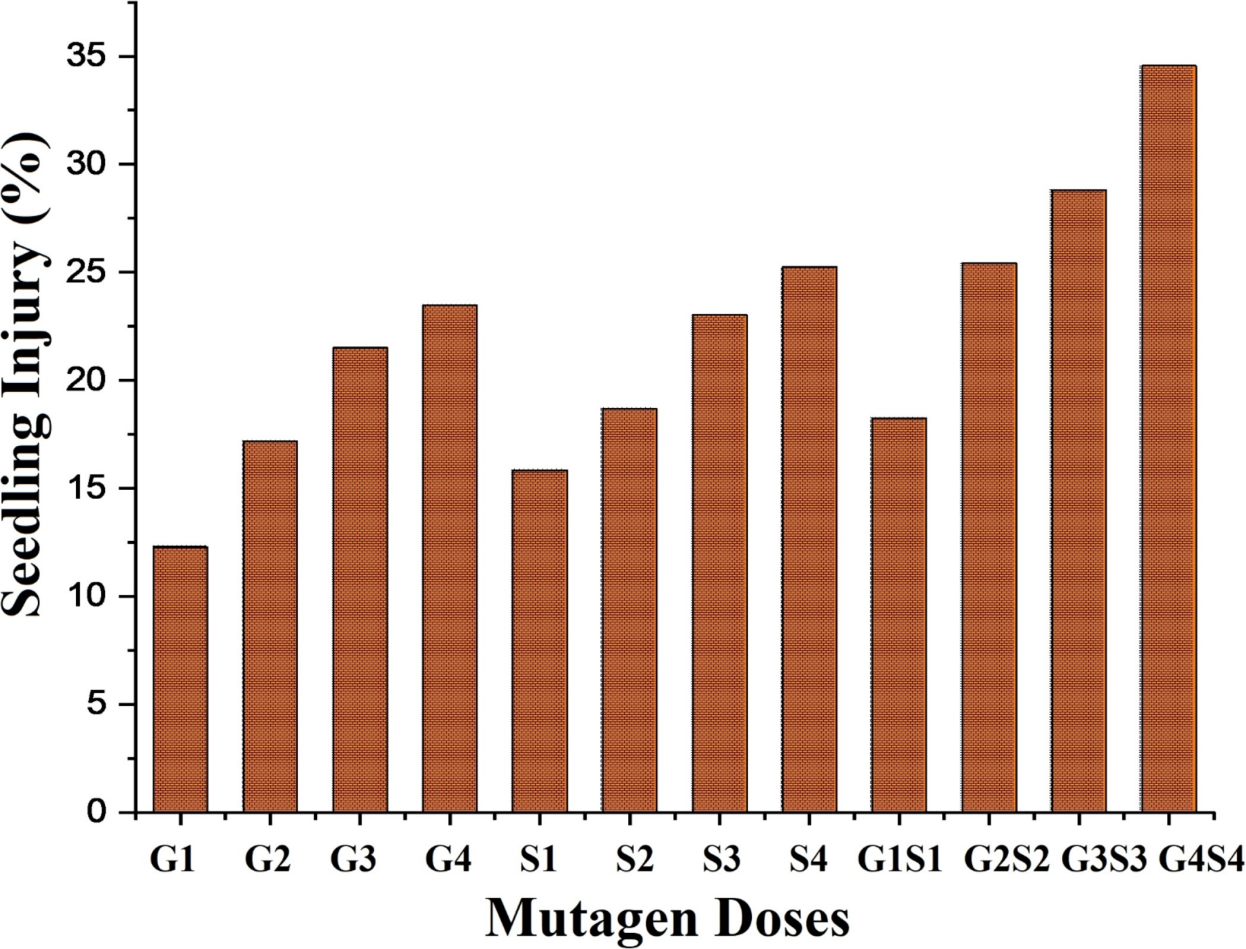
Effect of different doses of gamma rays sodium azide and their combinations on seedling injury (%) in var. Pusa-578. G1 = 100 Gy γ rays, G2 = 200 Gy γ rays, G3 = 300 Gy γ rays, G4 = 400 Gy γ rays, S1 = 0.01% SA, S2 = 0.02% SA, S3 = 0.03% SA, S4 = 0.04% SA, G1+S1 = 100 Gy γ rays+0.01% SA, G2+S2 = 200 Gy γ rays+0.02% SA, G3+S3 = 300 Gy γ rays+0.03% SA, G4+S4 = 400 Gy γ rays+0.04% SA.

The minimum seedling injury was recorded as 10.91 and 12.28% in 100 Gy treatment in the var. Gomati VU-89 and var. Pusa-578, respectively (Figs 8 and 9).

### 3.2. Pollen fertility

The results revealed a statistically significant decrease in pollen fertility in all the mutagenized plants of both varieties. In control, the pollen fertility was recorded as 95.30 and 96.53% in the varieties Gomati VU-89 and Pusa-578, respectively (Table 2). While comparing with control, the minimum decrease in pollen fertility was recorded in 0.01% SA and 100 Gy γ rays treatment in the var. Gomati VU-89 (89.36%) and var. Pusa-578 (87.13%), respectively. In γ irradiated plants, the maximum pollen fertility was recorded in 400 Gy γ rays treatment in the var. Gomati VU-89 (16.36%) and var. Pusa-578 (21.79%). In SA treated plants, the maximum pollen fertility was recorded in 0.01% SA treatment in the var. Gomati VU-89 (22.07%) and var. Pusa-578 (24.36%). In combination treatments, the maximum pollen fertility was recorded in 400 Gy γ rays+0.04% SA treatment in the var. Gomati VU-89 (24.67%) and var. Pusa-578 (27.95%).

**Table 2:** Effects of gamma rays, sodium azide and their combinations on mean pollen fertility and pollen fertility reduction in two cowpea varieties. The data is presented as percent (n = 3) and standard error (SE). Mean values with the same letters are not significant at a 5% level of significance, based on Duncan’s multiple range test (DMRT).

**Treatment**	**Pollen fertility**	
	**Mean ± SE**	**% age reduction**
	**Gomati VU-89**
**Control**	95.30^a^±0.44	**0.00**
100Gy γ rays	89.11^b^±0.59	6.49
200Gy γ rays	86.88^c^±0.57	8.83
300Gy γ rays	84.65^d^±0.42	11.16
400Gy γ rays	79.70^f^±0.40	16.36
0.01% SA	89.36^b^±0.55	6.23
0.02% SA	85.15^d^±0.60	10.64
0.03% SA	80.20^ef^±0.57	17.03
0.04% SA	74.26^g^±0.44	22.07
100Gy γ rays+ 0.01% SA	89.11^b^±0.55	6.49
200Gy γ rays+0.01% SA	84.16^d^±0.53	11.68
300Gy γ rays+0.03% SA	81.68^e^±0.51	18.18
400Gy γ rays+0.04% SA	71.78^h^±0.48	24.67
**Treatment**	**Pusa-578**
**Control**	**96.53** ^**a**^ **±0.53**	**0.00**
100Gy γ rays	87.13^b^±0.51	9.74
200Gy γ rays	85.40^c^±0.55	11.53
300Gy γ rays	79.21^e^±0.54	17.94
400Gy γ rays	75.50^f^±0.53	21.79
0.01% SA	86.63^bc^±0.59	10.25
0.02% SA	82.92^d^±0.53	14.10
0.03% SA	75.74^f^±0.57	21.53
0.04% SA	73.02^g^±0.55	24.36
100Gy γ rays+ 0.01% SA	85.40^c^±0.57	11.53
200Gy γ rays+0.01% SA	81.93^d^±0.58	15.12
300Gy γ rays+0.03% SA	75.50^f^±0.55	21.79
400Gy γ rays+0.04% SA	69.55^h^±0.50	27.95

G1 = 100 Gy γ rays, G2 = 200 Gy γ rays, G3 = 300 Gy γ rays, G4 = 400 Gy γ rays, S1 = 0.01% SA, S2 = 0.02% SA, S3 = 0.03% SA, S4 = 0.04% SA, G1+S1 = 100 Gy γ rays+0.01% SA, G2+S2 = 200 Gy γ rays+0.02% SA, G3+S3 = 300 Gy γ rays+0.03% SA, G4+S4 = 400 Gy γ rays+0.04% SA

### 3.3. Cytological aberrations

**Fig 10:**
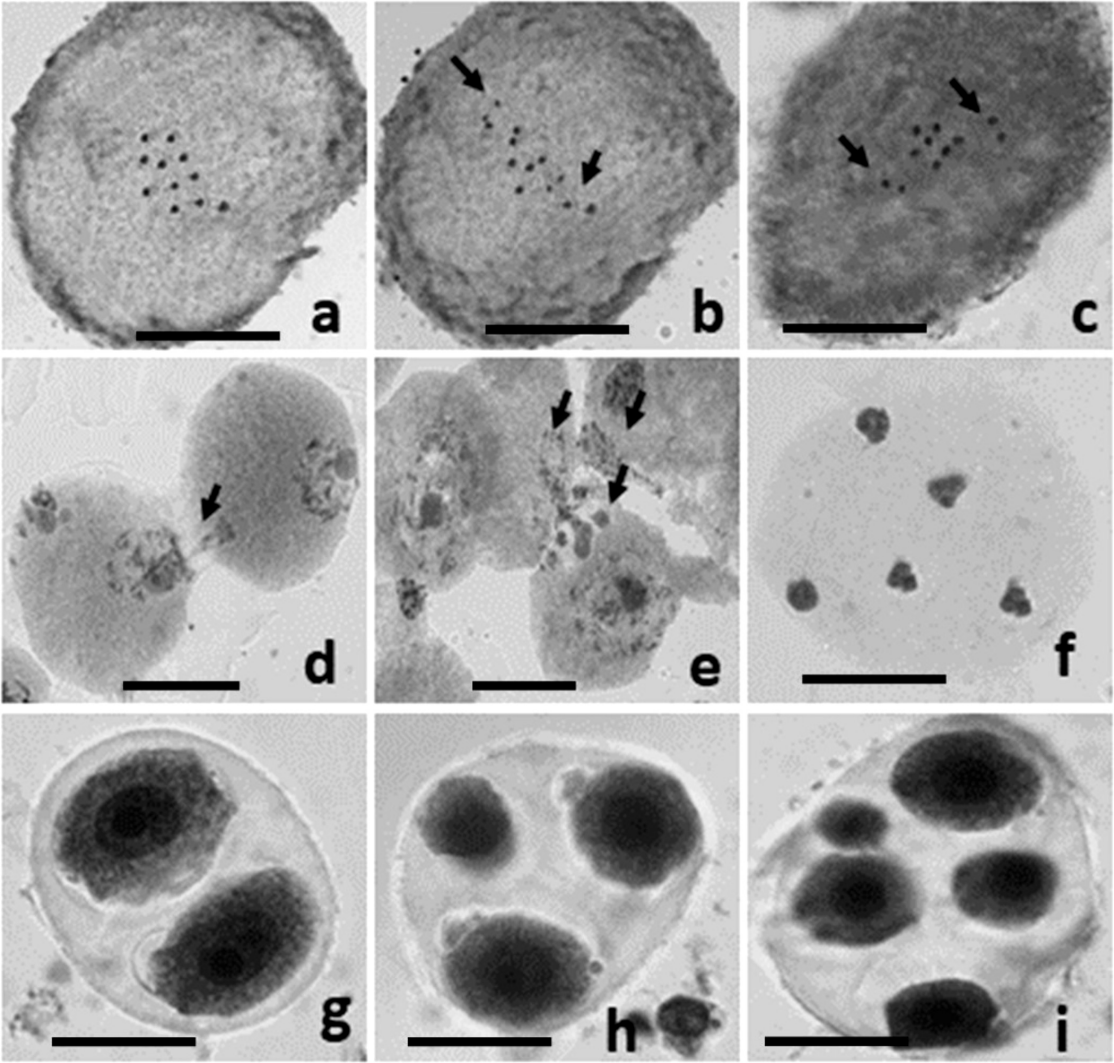
PMCs showing meiotic abnormalities in the treated population of cowpea **a:** PMC showing 11 bivalents at Metaphase-I (Control). **b:** PMC showing univalents at Metaphase-I (arrowed). **c:** PMC showing precocious separation at Metaphase-I (arrowed). **d:** Two PMCs involved in cytomixis at early prophase (arrowed). **e:** Three PMCs involved in cytomixis at early prophase (arrowed). **f:** PMC showing disturbed polarity at Telophase-II. **g:** Dyad **h:** Triad **i:** Polyad. Scale bar = 10μm.

Normal meiotic division with the formation of eleven perfect bivalents at metaphase-I followed by the normal separation (11:11) at anaphase-I was observed in the PMCs of floral buds from control plants. However, detailed meiotic analysis of the plants raised from treated seeds of both varieties showed a broad spectrum and a higher frequency of different chromosomal aberrations such as univalents, precocious separation of bivalents, bridges, laggards, stickiness, disturbed polarity, cytomixis, and abnormal sporads in the form of dyads, triads, and polyads at different stages of meiosis. Both varieties reflected almost similar spectrum of meiotic abnormalities with different frequencies at different doses of mutagens (Tables 3 and 4; Fig 10). Generally, a dose-dependent increase in meiotic aberrations was recorded in single and combination doses of γ rays and SA in both varieties. The frequency of meiotic aberrations was relatively more in the var. Gomati VU-89 as compared to the var. Pusa-578 thereby reflected the differential sensitivity of varieties toward mutagenic doses. Among the various chromosomal aberrations studied, the maximum frequency was recorded for chromosome stickiness in the variety Gomati VU-89 (1.37%) and Pusa-578 (1.16%). In contrast, the minimum frequency was recorded for polyad in the var. Gomati VU-89 (0.18%) and precocious separation in the var. Pusa-578 (0.18%). The highest total frequency of meiotic anomalies was recorded in combined γ rays + SA treatments, followed by γ rays and SA treatments in both varieties of cowpea. Different cytological aberrations observed in the present investigation are discussed as follows:

**Table 3:** Frequency of cytological aberrations in various mutagen treatments in cowpea var. Gomati VU-89.

	Frequency (%) of abnormal PMCs at different stages of meiosis													
Treatment	Total PMCs Observed	Prophase I/II	Metaphase I/II			Anaphase I/II		Telophase I/II					Total No. of Abnormal PMCs	Total frequency (%)
		C	U	CS	PS	US	B	L	DP	Dy	Tr	Po		
**Control**	**480**	**-**	**-**	**-**	**-**	**-**	**-**	**-**	**-**	**-**	**-**	**-**	**-**	**-**
G1	470	0.21	0.43	1.06	0.21	0.21	0.00	0.21	0.00	0.21	0.43	0. 21	15	3.20^f^
G2	486	0.62	1.23	1.23	0.21	0.00	0.21	0.21	0.21	0.41	0.21	0. 62	25	5.14^e^
G3	475	0.63	1.05	1.26	0.00	0.63	0.21	0.63	0.21	0.21	0.63	0.00	26	5.47^de^
G4	482	1.04	0.83	1.45	0.41	0.83	0.62	0.83	0.83	0.83	0.41	0.41	41	8.51^bc^
**Total**	**1913**	**0.63**	**0.89**	**1.25**	**0.21**	**0.42**	**0.26**	**0.47**	**0.31**	**0.42**	**0.42**	**0.31**	**107**	**5.59**
S1	490	0.20	0.41	1.02	0.00	0.00	0.00	0.00	0.00	0.00	0.00	0.00	8	1.63^g^
S2	486	0.62	0.62	1.23	0.21	0.00	0.00	0.21	0.21	0.00	0.21	0.21	17	3.50^f^
S3	515	0.78	0.97	0.97	0.58	0.19	0.58	0.58	0.39	0.19	0.39	0.39	31	6.02^de^
S4	485	1.24	1.44	2.06	0.00	0.82	1.03	0.82	0.62	0.00	0.00	0.00	37	7.63^c^
**Total**	**1976**	**0.71**	**0.76**	**1.32**	**0.20**	**0.25**	**0.40**	**0.40**	**0.30**	**0.05**	**0.15**	**0.15**	**93**	**4.71**
G1+S1	496	0.60	0.81	1.41	0.81	0.20	0.20	0.00	0.60	0.40	0.20	0.20	27	5.44^de^
G2+S2	498	0.40	0.80	1.61	1.00	0.60	0.20	1.00	0.80	0.00	0.00	0.00	32	6.43^d^
G3+S3	475	0.63	1.05	1.47	0.84	1.05	0.63	0.84	1.05	0.63	0.42	0.42	43	9.05^b^
G4+S4	485	1.03	1.44	1.65	1.03	1.24	0.41	0.62	0.82	0.82	0.62	0.62	50	10.31^a^
**Total**	**1954**	**0.67**	**1.02**	**1.54**	**0.92**	**0.77**	**0.36**	**0.61**	**0.82**	**0.46**	**0.31**	**0.31**	**152**	**7.78**
**Grand Total**	**5843**	**0.67**	**0.89**	**1.37**	**0.44**	**0.48**	**0.34**	**0.50**	**0.48**	**0.31**	**0.29**	**0.26**	**352**	**6.02**

G1 = 100 Gy γ rays, G2 = 200 Gy γ rays, G3 = 300 Gy γ rays, G4 = 400 Gy γ rays, S1 = 0.01% SA, S2 = 0.02% SA, S3 = 0.03% SA, S4 = 0.04% SA, G1+S1 = 100 Gy γ rays+0.01% SA, G2+S2 = 200 Gy γ rays+0.02% SA, G3+S3 = 300 Gy γ rays+0.03% SA, G4+S4 = 400 Gy γ rays+0.04% SA, C = Cytomixis U = Univalent, CS = Chromosome stickiness, PS = Precocious separation, US = Unequal separation, B = Bridge, L = Laggard, DP = Disturbed polarity, Dy = Dyad, Tr = Triad, Po = Polyad.

**Table 4:** Frequency of cytological aberrations in various mutagenic treatments in cowpea var. Pusa-578.

	Frequency (%) of abnormal PMCs at different stages of meiosis													
Treatment	Total PMCs Observed	Prophase I/II	Metaphase I/II			Anaphase I/II		Telophase I/II					Total No. of Abnormal PMCs	Total frequency (%)
		C	U	CS	PS	US	B	L	DP	Dy	Tr	Po		
**Control**	**465**	**-**	**-**	**-**	**-**	**-**	**-**	**-**	**-**	**-**	**-**	**-**	**-**	**-**
G1	470	0.21	0.85	1.28	0.00	0.21	0.00	0.00	0.00	0.00	0.21	0. 43	15	3.19^d^
G2	468	0.64	0.64	0.85	0.21	0.21	0.64	1.07	0.00	0.64	0.43	0. 43	27	5.77^c^
G3	480	1.25	1.04	0.83	0.00	0.42	0.83	0.83	0.63	0.42	0.63	0.00	33	6.88^b^
G4	460	0.87	1.30	1.52	0.65	0.22	0.65	0.87	0.87	0.65	0.65	0.22	39	8.48^a^
**Total**	**1878**	**0.75**	**0.96**	**1.12**	**0.21**	**0.27**	**0.53**	**0.69**	**0.37**	**0.43**	**0.48**	**0.27**	**110**	**5.86**
S1	475	0.00	0.00	0.84	0.00	0.00	0.00	0.21	0.00	0.00	0.00	0.00	5	1.05^e^
S2	478	0.42	0.84	1.05	0.00	0.00	0.21	0.21	0.00	0.00	0.42	0.42	17	3.56^d^
S3	460	0.65	0.65	1.09	0.22	0.65	0.65	0.22	0.00	0.43	0.22	0.22	23	5.00^c^
S4	468	1.07	1.28	1.71	0.21	0.21	0.43	0.64	1.07	0.00	0.00	0.00	31	6.62^b^
**Total**	**1881**	**0.53**	**0.69**	**1.17**	**0.11**	**0.21**	**0.32**	**0.32**	**0.27**	**0.11**	**0.11**	**0.27**	**75**	**3.99**
G1+S1	460	0.65	0.87	1.74	0.22	0.00	0.00	0.65	0.00	0.65	0.43	0.00	26	5.65^c^
G2+S2	469	1.07	1.07	0.85	0.21	0.64	0.64	0.21	0.64	0.00	0.00	0.42	23	4.90^c^
G3+S3	462	0.65	0.43	1.08	0.22	0.43	0.43	0.65	0.22	0.43	0.22	0.22	26	5.63^c^
G4+S4	451	0.44	1.77	1.11	0.22	0.22	0.22	0.44	0.89	1.11	0.89	0.00	38	8.43^a^
**Total**	**1842**	**0.67**	**1.04**	**1.19**	**0.22**	**0.33**	**0.33**	**0.61**	**0.43**	**0.54**	**0.31**	**0.16**	**113**	**6.13**
**Grand Total**	**5601**	**0.66**	**0.89**	**1.16**	**0.18**	**0.30**	**0.39**	**0.50**	**0.36**	**0.36**	**0.32**	**0.27**	**298**	**5.32**

G1 = 100 Gy γ rays, G2 = 200 Gy γ rays, G3 = 300 Gy γ rays, G4 = 400 Gy γ rays, S1 = 0.01% SA, S2 = 0.02% SA, S3 = 0.03% SA, S4 = 0.04% SA, G1+S1 = 100 Gy γ rays+0.01% SA, G2+S2 = 200 Gy γ rays+0.02% SA, G3+S3 = 300 Gy γ rays+0.03% SA, G4+S4 = 400 Gy γ rays+0.04% SA, C = Cytomixis U = Univalent, CS = Chromosome stickiness, PS = Precocious separation, US = Unequal separation, B = Bridge, L = Laggard, DP = Disturbed polarity, Dy = Dyad, Tr = Triad, Po = Polyad

- Cytomixis: An abnormality involving chromatin transfer between adjacent PMCs termed cytomixis was observed in all mutagenized populations, with a progressive increase in the higher mutagen doses in both varieties. The chromatin transfer in cytomixis was either partial, with a small part of chromosomes in most of the PMCs, or complete involving an entire chromosome complement in a few PMCs. The maximum frequency of PMCs showing cytomixis was recorded in 0.04% SA and 400 Gy γ rays+0.04% SA treatments in the var. Gomati VU-89 (1.24%) and 300 Gy γ rays treatments in the var. Pusa-578 (1.25%) (Tables 3 and 4).
- Univalents: In both varieties, univalents at metaphase-I were observed frequently in combination treatments followed by γ rays and SA. The maximum frequency of PMCs showing univalents was recorded in 0.04% SA and 400 Gy γ rays+0.04% SA treatments in the var. Gomati VU-89 (1.44%) and Pusa-578 (1.77%), respectively (Tables 3 and 4).
- Chromosome Stickiness: The highest frequency of chromosome stickiness, a compact chromatin mass type at metaphase-I, was recorded in combination treatments followed by SA and γ rays. The maximum frequency of PMCs showing chromosome stickiness was recorded in 0.04% SA and 400 Gy γ rays+0.04% SA treatments in the var. Gomati VU-89 (2.06%) and Pusa-578 (1.74%), respectively (Tables 3 and 4).
- Precocious Separation: Higher doses of combined mutagens induced a maximum precocious separation of chromosomes to the opposite poles compared to the individual mutagen doses. The maximum frequency of PMCs showing precocious separation was recorded in 400 Gy γ rays+0.04% SA and 400 Gy γ rays treatments in the var. Gomati VU-89 (1.03%) and Pusa-578 (0.65%), respectively (Tables 3 and 4).
- Unequal Separation: The maximum frequency of PMCs showing unequal separation was recorded in 400 Gy γ rays+0.04% SA and 0.03% SAtreatments in the var. Gomati VU-89 (1.24%) and Pusa-578 (0.65%), respectively (Tables 3 and 4).
- Chromosome Bridges: The chromosome bridges observed at anaphase I was mostly of a single type at a very low frequency in lower mutagen doses. The maximum frequency of PMCs showing chromosome bridges was recorded in 0.04% SA and 300 Gy γ rays and G3 treatments in the var. Gomati VU-89 (1.03%) and Pusa-578 (0.83%), respectively (Tables 3 and 4).
- Laggards: The maximum frequency of PMCs showing laggards was recorded in 200 Gy γ rays+0.02% SA and 200 Gy γ rays treatments in the var. Gomati VU-89 (1.00%) and Pusa-578 (1.07%), respectively (Tables 3 and 4).
- Disturbed Polarity: The maximum frequency of PMCs showing disturbed polarity was recorded in 300 Gy γ rays+0.03% SA and 0.04% SA treatments in the var. Gomati VU-89 (1.05%) and Pusa-578 (1.07%), respectively (Tables 3 and 4).
- Dyads, Triads and Polyads: In addition to these abnormalities, dyads and triads were also observed in all the mutagen doses except 200 Gy γ rays+0.02% SA treatment. However, polyads were observed in all the mutagens treatments in both varieties. The maximum frequency of PMCs showing dyads was recorded in 400 Gy γ rays and 400 Gy γ rays+0.04% SA treatments in the var. Gomati VU-89 (0.83%) and Pusa-578 (1.11%), respectively (Tables 3 and 4). The maximum frequency of PMCs showing triads was recorded in 300 Gy γ rays and 400 Gy γ rays+0.04% SA treatments in the var. Gomati VU-89 (0.63%) and Pusa-578 (0.89%), respectively (Tables 3 and 4). The maximum frequency of PMCs showing polyads was recorded in 200 Gy γ rays and 400 Gy γ rays+0.04% SA in the var. Gomati VU-89 (0.62%) and 100 Gy γ rays and 200 Gy γ rays treatments in Pusa-578 (0.43%) (Tables 3 and 4).

## 4. Discussion

### 4.1. Evaluation of LD50

In the mutation breeding programs balance between desirable mutations and mortality or death of seedlings forms an important component in achieving the aims of the study. A dose that induces maximum frequency of mutations and minimum damage or mortality is considered an optimum dose. A lot of research has already been conducted in determining the optimum dose. A collective view about the doses is that the dose that kills 50% of seedlings is termed LD50, and any dose below LD50 might yield good results [32, 33]. Therefore, assessing LD50 holds a place of paramount importance in all mutation breeding programs. LD50 provides an idea about a good balance between biological damage in terms of lethality and frequency of desirable mutations. In the present study, a minor difference in the values of LD50 doses for γ rays and SA was observed in the var. Gomati VU-89 and var. Pusa-578. The results were in propinquity with the findings of Sao *et al*. [34], who also reported differences in the LD50 values of γ ray, X rays, proton beam and electron beam in two rice varieties. Kumar *et al*. [35] also reported a difference in LD50 values in linseed varieties treated with ethyl methane sulphonate and γ rays. The difference in the LD50 values of the same mutagen may be attributed to differences in genetics, and physiology of the varieties. It also depends on other factors, such as moisture content of seeds and the recovery mechanism after mutagen exposure. LD50 values were less in Gomati VU-89, indicating it was more sensitive to γ rays than var. Pusa-578. In both varieties, germination decreased progressively as the mutagen dose increased, which is obvious as higher mutagen doses are more detrimental compared to lower doses.

### 4.2. Bio-physiological damages

The study on mutagen induced bio-physiological and cytological aberrations is considered as one of the most reliable index to determine the genotypic sensitivity and mutagenic potency, which is imperative for optimizing a mutagen dose.

#### 4.2.1. Seed germination and seedling growth parameters

In the present study, a considerable reduction in seed germination in the treated population was in accordance with the findings of Raina *et al*. [36], who reported a reduced seed germination in mutagenized chickpeas. Inhibition of metabolic activation or damage of cell constituents may be attributed to reduced seed germination in treated populations. Combined mutagens induced maximum germination inhibition may be attributed to the synergistic effect of the two mutagens. Mutagens while interacting with the seed tissues, cause disruption and disorganization of the tunica layer, which may be another probable reason for decreased seed germination in treated populations. Similarly, mutagens induced decrease in seedlings height in the present study was in accordance with the findings of Taziun *et al*. [24], who reported a proportionate decrease in seedling height in mutagen-treated lentil populations. In the present study, combined mutagens induced a maximum reduction in seedling height compared to individual treatments of γ rays and SA, which might be attributed to a synergistic effect of the two mutagens. The probable reasons for the seedling injury may be attributed to the mutagen induced chromosomal damages and/or inhibition of cell proliferation. Mutagen induced alteration in enzyme activities and bio-physiological processes in the seeds and seedlings might also be attributed to decreased seedling height in mutagen treated populations.

### 4.3. Pollen fertility

As expected, we noticed a progressive decline in the pollen fertility percentage with the increase in the mutagen doses. The results of the present study were in propinquity with the findings of previous studies in various crops such as garden beans [37], lentils [24], [38], horse gram [39], and pigeon pea [40]. The normal segregations of chromosomes without any structural alterations are important in developing normal pollen grains. The reduced pollen fertility in the plants treated with higher doses of mutagens may be due to chromosomal aberrations that also occurred at higher frequencies in such plants. The earlier studies also supported this view that reduced pollen sterility occurs due to mutagen-induced chromosomal anomalies and hence genetic alterations resulting in aberrant pollen grains [20, 41, 42].

### 4.4. Cytological aberrations

The meiotic cycle is an important process associated with any organism’s cellular division and propagation; any alteration in its regulation affects survival. It is necessary for cells to follow a correct meiotic path and result in full gametic viability. Several genes act pre and post-meiotically to control the cytological stages of the cell cycle in germ cells. Mutagen induced alterations in any of these genes are deleterious and result in severe meiotic abnormalities that may impact the species viability and overall reproductive capacity [43–46]. Meiotic abnormalities lead to the formation of chromosomally imbalanced gametes, which ultimately affect the pollen and seed viability as stated previously by several workers [47, 48]. During the present investigation, many meiotic abnormalities such as cytomixis, univalent formation, chromosome stickiness, precocious separation, unequal separation, bridges, laggards, disturbed polarity, dyads, triads, and polyads have been observed in the course of meiosis in most of the analyzed PMCs. A dose-dependent increase in the frequency of meiotic abnormalities was in good agreement with the findings of Khursheed *et al*. [49], Amin *et al*. [50], Bhat *et al*. [51] who reported maximum meiotic abnormalities at higher doses of mutagens. Similarly, the maximum frequency of meiotic aberrations recorded in the combined mutagen treatments followed by individual treatments of γ rays and SA was in accordance with the findings of Goyal *et al*. [52], who reported maximum meiotic abnormalities in PMCs of urdbean treated with combined mutagen treatments.

In the present study, cytomixis, an abnormality in which genetic material is transferred from cell to cell through single and multiple cytoplasmic connections at prophase-I/II, has been noticed in high frequency in PMCs of treated populations in both varieties. It indicated that mutagen treated plants may be screened for varied ploidy levels and their utilization in plant breeding programs may be pursued. Cytomixis may lead to the formation of hypo-/hyperploid cells and uneven gametes that could play a vital role in chromosomal diversity and the evolution of aneuploids and polyploids [53]. Mutagens have been reported as factors that lead to the formation of cytomixis in mutagenized populations [51]. Not only mutagens, sublethal artifacts produced by the fixation and/or mechanical injury may also induce the formation of cytomixis. The appearance of cytomixis has also been reported in other non-mutagenized plants [48, 54–57]. Earlier workers have reported that cytomixis may be induced by several factors, such as temperature [58], the combined role of stress and genetic factors [59], and direct genetic control [60, 61].

Univalents occurred at metaphase-I, and their frequency was higher in combination treatments of γ rays and SA. The univalent is either a chromosome that fails to pair at zygotene and remains separated at diplotene due to the lack of chiasma formation or the precocious separation of bivalents at anaphase. The results were in accordance with the reports of Amin *et al*. [50] that showed a maximum frequency of univalents in black cumin mutants treated with higher doses of mutagens. Previously it has been reported that the occurrence of univalents in the mutagenized populations may be due to slipping off of the chiasmata or absence of pairing that lead to failure of chromosome movement and disturbed normal pairing of homologous chromosomes [49, 62]. Mutagens induced restricted pairing between homologous chromosomes and decreased chiasma formation may also be attributed to univalent formation in mutagenized populations.

In the present study, chromosome stickiness, an abnormality associated with the chromosome clumping, was observed in high frequency in treated populations. The chromosome stickiness delays the normal separation of bivalents and lead to the formation of laggards, chromatin bridges, micronuclei, and sterile pollen grains. Mutagen induced chromosome stickiness in faba bean mutants treated with γ rays and ethyl methane sulphonate has been reported by Khursheed et al. [49]. Nucleic acid depolymerization or partial nucleoprotein dissociation could also lead to chromosome stickiness [63]. It may be attributed to mutagen induced malfunctioning of certain genes linked with meiosis. It may also be due to gene mutations that lead to incorrect coding of non-histone proteins involved in chromatid segregation during active divisional stages. In addition to mutagens action, stickiness could also be induced by other factors such as soil elements [64, 65], herbicides [66], and soil chemicals [67, 68].

The cytological phenomenon where homologous chromosomes pair showed a lack of synchrony in their segregation at metaphase-I or anaphase-I is known as non-synchronous disjunction. The non-synchronous disjunction includes precocious and late disjunction/unequal separation of chromosomes. Non-synchronous disjunction can arise spontaneously [69] or be induced by different mutagens [70, 71]. Precocious chromosome separation has been noticed in high frequency in mutants treated with higher doses of γ rays and SA. The results were in accordance with the findings of Bhat and Wani. [72], who reported an increased frequency of precocious separation of chromosomes at higher doses of mutagens in faba bean mutants. In the current study, the precocious separation may be attributed to mutagen induced alterations in the formation and functioning of chiasmata required for the maintenance of bivalents and normal chromosome segregation. In the present study, the maximum frequency of unequal separation has been noticed in combined treatments followed by γ rays and SA. The results were in accordance with the findings of Amin *et al*. [50], who reported a higher frequency of unequal separation in combined mutagen treatments in black cumin cultivars. Non-synchronous disjunction may be due to chiasmata interlocking, lack of coordination between spindle and chromosomes [73], varied rates of terminalisation [74, 75], altered chromosome homology [76]. The disturbed orientation and separation of bivalents resulted in chromatin bridges, laggards and unequal distribution of chromosomes at poles during anaphases, ultimately affecting pollen fertility.

The chromatin bridge formation was observed for the first time by Greet in 1911 [77]. A lengthy chromosomal bridge known as the chromatin bridge is created during cell division when a chromosome containing two centromeres is dragged to the opposite poles of the cell. Several authors have provided explanations for the origins of bridge formation, including paracentric inversion [78], failure of chiasmata in a bivalent to terminalize [79], and interlocking of bivalent chromosomes [80]. The chromosome bridges were observed at anaphase I in the treated population with maximum frequency at the highest doses of mutagens. Bhat and Wani [72] also reported bridges with maximum frequency at the highest doses of combination and individual mutagenic treatments in faba bean mutants. The bridge formation may be attributed to the sister chromatid exchange followed by delayed or no separation at subsequent stages. Mutagen induced formation of anaphase bridges may also be attributed to the chromosome breakage followed by a reunion of broken ends [81]. El-Ghamery *et al*. [82] also believe that breakage and reunion of these chromosomes lead to the formation of dicentric chromosomes that result in the formation of single and multiple bridges. In the present study, the occurrence of chromosome bridges at anaphase-I could be due to stickiness of chromosomes at metaphase I, which restrict their movement towards opposite poles.

The chromosomes or bivalents that stay in the middle and fail to spread at their corresponding poles at the anaphase or telophase stage lead to the formation of laggards. The PMCs with laggards were observed in almost all the mutagen treatments, with the maximum frequency in higher doses of mutagens. The results were in accordance with the findings of Bhat and Wani [72], who reported an increased frequency of laggards at higher mutagen doses. Delayed terminalisation, abnormal spindle formation, and failure of chromosomal movement may also be attributed to the formation of laggards.

To ensure that mitosis and meiosis proceed normally and, ultimately that species survive, spindle formation and function must be coordinated with other nuclear division processes [83]. A balanced genetic makeup and the creation of viable gametes depend on forming a bipolar spindle [84]. Any irregular spindle activity results in disturbed polarity, which can lead bivalents out of orientation and, as a result, subgroups of chromosomes that perform independently appear [85]. The increased frequency of PMCs with disturbed polarity at higher doses of mutagens was in agreement with the findings of Khursheed *et al*. [49], who reported increased disturbed polarity at higher mutagen doses. The disturbed polarity at telophase-II stages may be due to mutagen induced spindle disturbances in treated populations.

The abnormal microsporogenesis is directly correlated with the aberrant meiotic course observed presently and such irregular microsporogenesis results in abnormal sporads such as dyads, triads, and polyads. The formation of abnormal sporads at telophase-I/II stages was found in all mutagen treatments except 200 Gy γ rays+0.02% SA in both varieties. Several workers have also reported the formation of dyads, triads, and polyads in Tuberosum [86, 87] potato [88] and roses [89]. The lack of cytokinesis in meiosis II may be attributed to the formation of dyads with two diploid cells and triads with two haploid cells and one diploid cell [90].

## 5. Conclusions

The study revealed the importance of having a prior knowledge of mutagen induced bio-physiological damages and cytological aberrations for selecting optimum mutagen dose. A progressive decline in seed germination and seedling height with the increasing dose of γ rays and SA indicated the deleterious effects of higher mutagen doses on the crop physiology. Combined treatments induced a higher frequency of meiotic abnormalities followed by γ rays and SA, and therefore combined mutagen treatments should be avoided in further mutation breeding programs. This study also revealed varietal sensitivity toward mutagen dose, var. Gomati VU-89 showed a higher frequency of meiotic abnormalities than the var. Pusa-578, thereby reflecting a differential mutagenic sensitivity among varieties of diverse origin. This was also shown by the less LD50 values of both gamma rays and sodium azide in var. Gomati VU-89 compared to var. Pusa-578.
